# Mastalgia as an atypical presentation of hepatocellular carcinoma: a case report

**DOI:** 10.1186/s12957-017-1133-4

**Published:** 2017-03-09

**Authors:** AbdulAziz Mohammad Al-Sharydah, Abdulrhman Hamad Al-Abdulwahhab, Ibrahim Abobaker Alghnimi, Mohammed A. El Shawarby, Faisal Ahmad Katbi

**Affiliations:** 1 Department of Radiology, King Fahd Hospital of the University, University of Dammam, P.O. Box 4398, 31952 Dammam, Eastern Province Saudi Arabia; 2 Department of Pathology, King Fahd Hospital of the University, University of Dammam, Dammam, Eastern Province Saudi Arabia; 3 Department of Emergency Medicine, King Fahd Hospital of the University, University of Dammam, Dammam, Eastern Province Saudi Arabia

**Keywords:** Metastatic hepatocellular carcinoma, Extrahepatic, Bone metastasis, Liver cancer

## Abstract

**Background:**

As the incidence of hepatocellular carcinoma (HCC) diagnoses in Saudi Arabia has recently increased due to better diagnostic techniques, the incidence of diagnosed HCC metastasis has also increased. Here, we report a case of HCC metastasis to the rib with an initially atypical presentation of mastalgia caused by extrahepatic metastasis.

**Case presentation:**

A 31-year-old woman with a prior hepatitis B viral infection presented with a mass in the left breast accompanied by mastalgia for a 6-month duration. The patient’s liver enzymes were elevated, and her serum α-fetoprotein level was particularly high. Computed tomography of her chest and abdomen showed a soft-tissue mass adhering to the upper chest wall, rib deterioration, and multiple hepatic lesions. A needle biopsy was immunohistochemically analyzed for Glypican-3, Pan-CK, and CK7 and was confirmed to be metastatic HCC.

**Conclusions:**

This metastatic HCC case is unique because it initially presented as mastalgia. We should consider the possibility of metastatic disease when assessing patients with unusual presentations who have risk factors for metastatic carcinoma.

## Background

Hepatocellular carcinoma (HCC) is the 3rd most common carcinoma worldwide. In Saudi Arabia, it accounts for approximately 4.8% of all tumors. HCC is the 4th most common cancer in men and the 8th most common cancer in women [1] and is defined as an epithelial tumor that arises from the malignant transformation of hepatocytes. HCC is the most common primary liver malignancy and is often discovered in patients who present with cirrhosis or other chronic liver diseases [2]. Extrahepatic metastasis (Em) of HCC (EmHCC) is usually seen in a terminal stage and has an aggressive progression [3]. EmHCC manifests in approximately half of HCC cases and is most commonly found in the lung, lymph nodes, or bones [4]. Bone metastasis accounts for 1.6–16% of HCC metastasis. Em occurs most commonly to the ribs (six reported cases), followed by the mandible, the humerus, the femur, the iliac bone, and the vertebral bodies [5, 6]. The prognosis of Em is poor, with a 1-year survival rate of approximately 40% [7–9]. In this report, we present an unusual metastatic HCC case that initially presented as mastalgia caused by an EmHCC to the rib cage.

## Case presentation

A 31-year-old housewife of African descent with a prior history of chronic hepatitis B infection and hypothyroidism presented with a mass in her left breast and mastalgia lasting for 6 months. Her vital signs were normal. Upon physical examination, a non-mobile, round mass of approximately 5 × 6 cm was palpated in the left breast. The rest of the exam was normal, and assessment of other clinical parameters showed nothing unusual. However, laboratory findings showed an elevated serum α-fetoprotein (AFP) level of 402.5 ng/cc and elevated alanine transaminase and aspartate transaminase levels of 94 and 651 IU/L, respectively (Table 1).

**Table 1:** Biochemical test results and tumor marker levels

	Test	Result (normal range)
CBC	Hemoglobin	11.8 (13.0–18.0) g/dL
	WBC count	8.7 (4.0–11.0) k/μL
	Platelet	150 (140–450) k/μL
LFT	Serum total protein	6.1 (6–8) g/dL
	Albumin	2.3 (3.5–5) g/dL
	SGOT	651 (15–37) U/L
	SGPT	94 (14–63) U/L
	LDH	726 (81–234) U/L
	GGTP	376 (5–55) U/L
	ALP	238 (46–116) U/L
Tumor markers	AFP	402.5 (0–40) ng/mL
	BHCG	1.20 (5–25) mlU/mL
	CA 125	27.7 (<35) U/mL
	CA 19-9	605.57 (0–37) U/mL
	CEA	0.50 (≤3) ng/mL

**Fig. 1:**
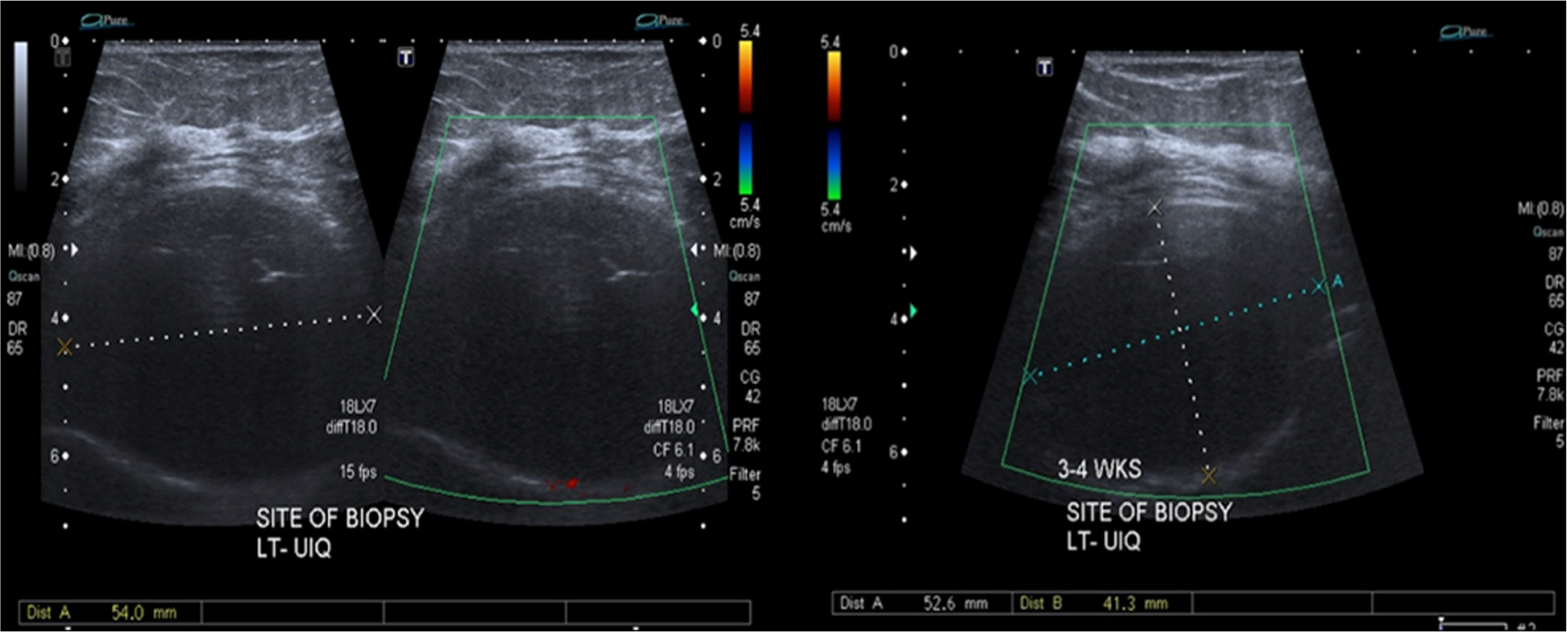
Grayscale and Doppler ultrasound of the left breast exhibit a large (5.2 × 4.2 × 5.4 cm) hypoechoic/anechoic lesion located at the left retro-pectoralis region with no evidence of vascularity

**Fig. 2:**
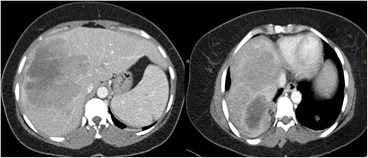
Transaxial images of the contrast-enhanced CT scan of the abdomen at the portal venous phase shows a multifocal, ill-defined, and relatively hypodense hepatic lesion that involves the right and left hepatic lobes and surrounds an enhanced pseudocapsule. There are multiple subcapsular undulated margins, which are suggestive of subcapsular retraction. The lesions show mass effects on the intrahepatic portion of the IVC with significant narrowing and medial deviation. The indistinct appearance of the middle hepatic vein and the distal branches of the portal vein suggest that HCC invaded these areas

**Fig. 3:**
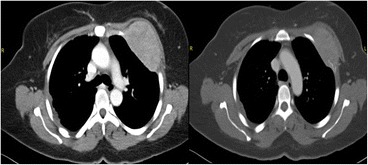
Transaxial images of the contrast-enhanced CT scan of the chest. *Left*: mediastinal window. *Right*: bone window shows an aggressive, destructive bone lesion with a large soft-tissue component arising from the 3rd anterolateral rib, which invaded the adjacent pleural surfaces and the ipsilateral major pectoral muscle

Although diagnostic mammography findings were negative, a breast examination showed a retrograndular versus submuscular hypoechoic mass measuring 5.2 × 4.2 × 5.4 cm in the left breast (Fig. 1). Consequently, the patient received a contrast chest and abdomen CT, which showed multifocal hypodense hepatic lesions with centralized necrosis and a tumoral thrombus in the intrahepatic portal vein (Fig. 2). A large, solitary mass with a large soft-tissue component was also seen on one of the patient’s left ribs, which was accompanied by deterioration of the patient’s 3rd rib bone. The mass bordered the ipsilateral pectoralis muscle, and it is likely that there was localized invasion (Fig. 3).

**Fig. 4:**
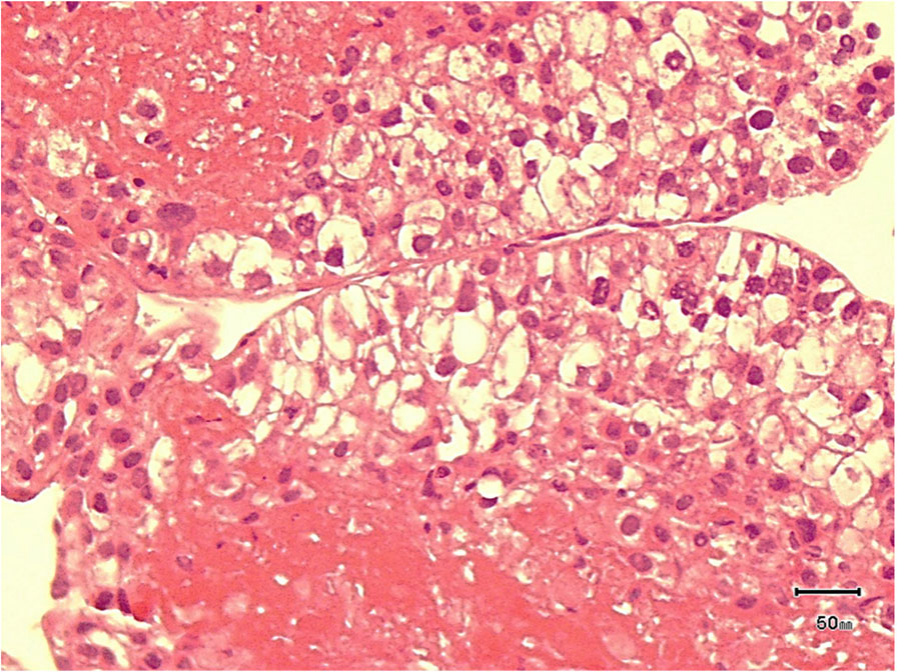
Higher magnification show the details of the cancer cells, which are arranged in a trabecular sinusoidal pattern with a clear cytoplasm and pleomorphic nuclei. Mitoses are seen in some areas, as are some foci of necrosis

**Fig. 5:**
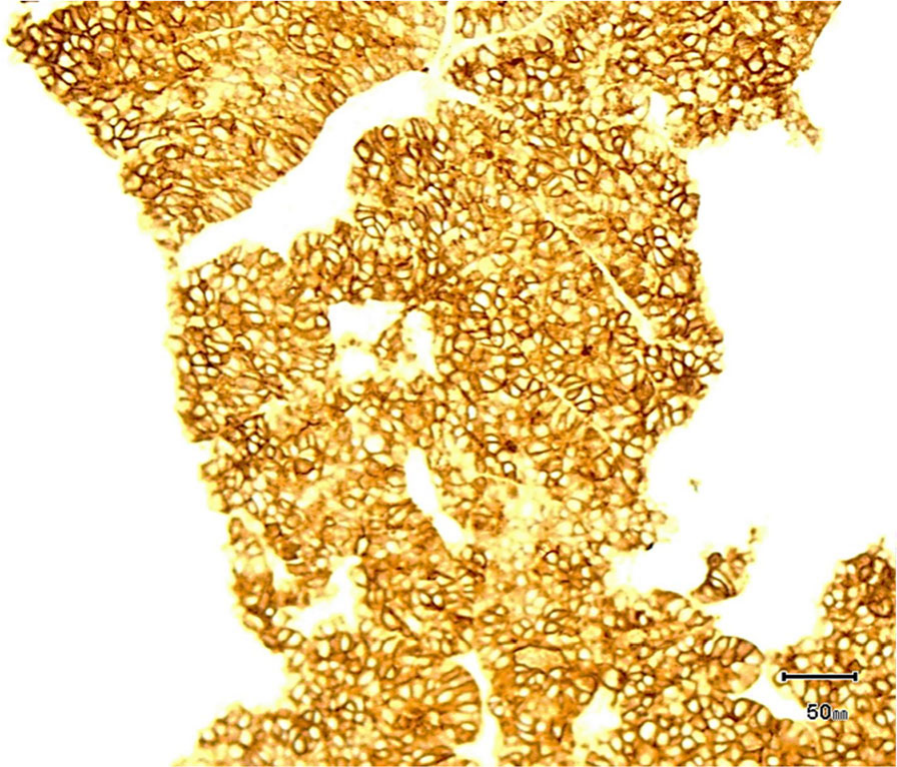
Bone with hepatocellular metastases; the *brown staining* is positive for glypican 3, which is specific for HCC

The patient subsequently received an image-guided needle biopsy of the mass. The biopsy specimen was subjected to specialized immunohistochemical staining (Fig. 4). The pathology report confirmed a metastatic epithelial tumor with morphological features that were hepatic in origin (Fig. 5).

## Discussion

Accurate diagnosis is the 1st step in optimal cancer care. However, because EmHCC often presents atypically, patients might be misdiagnosed and not receive appropriate treatment in a timely manner. Moreover, distinguishing a primary cancer from secondary metastatic lesions can be challenging. Fortunately, the evolution of diagnostic imaging modalities can assist the physician in accurate diagnostic practices.

In our study, a contrast-enhanced CT scan of the chest showed a destructive lesion on the left 3rd rib as a large soft-tissue mass that anteriorly invaded the ipsilateral pectoralis muscle, causing localized invasion of the breast tissue. Moreover, a contrast-enhanced CT scan of the abdomen showed multifocal hypodense hepatic lesions with centralized necrosis that surrounded an enhanced pseudocapsule and distal branches of the portal vein, all of which are features suggestive of HCC [10].

Nevertheless, a noninvasive approach to diagnose EmHCC might not be sufficiently precise. Undoubtedly, immunohistochemical diagnosis from needle aspiration biopsy is the gold standard because it provides details about the neoplastic cells involved in the malignant disease process (e.g., arrangement pattern and stain confirmation). Our patient was diagnosed with stage IV HCC with a metastatic epithelial tumor to the 3rd rib. This diagnosis was confirmed by specialized immunohistochemical stains including Glypican-3, cytokeratin pan-antibody (Pan-CK), and cytokeratin 7 (CK7). Glypican-3 is a membrane-bound heparin sulfate proteoglycan that is expressed only in HCC cells and their metastases (Fig. 5) [11].

Interestingly, hepatocytes can grow in trabecular structures that are surrounded by tissue expressing high levels of ECM proteins such as laminin, vitronectin, collagen, and fibronectin [12]. The defective cell adhesion and migration and proteolysis of extracellular matrix proteins are necessary steps in metastatic development [13]. However, EmHCC can be disseminated either hematogenously or lymphatically to form new neoplastic foci in sites other than the original tumor [14].

The clinical course and prognosis of EmHCC are not entirely clear. However, recently developed management options can prolong the life expectancy of HCC patients. Furthermore, systemic therapy with sorafenib combined with 5-fluorouracil has shown promising effects for HCC treatment [15].

## Conclusions

In summary, although EmHCC diagnosis can be challenging, physicians should be alert to the possibility of encountering this disease in their differential diagnoses. Regardless of AFP levels, EmHCC requires meticulous evaluation to reach an appropriate diagnosis and to provide suitable treatment.

## Abbreviations

AFP: Alpha fetoprotein
CK7: Cytokeratin 7
Em: Extrahepatic metastasis
EmHCC: Extrahepatic metastasis of hepatocellular carcinoma
HCC: Hepatocellular carcinoma
Pan-CK: Cytokeratin pan-antibody
